# Cancer literacy differences of basic knowledge, prevention, early detection, treatment and recovery: a cross-sectional study of urban and rural residents in Northeast China

**DOI:** 10.3389/fpubh.2024.1367947

**Published:** 2024-05-14

**Authors:** Mengdan Li, Ping Ni, Tingting Zuo, Yunyong Liu, Bo Zhu

**Affiliations:** ^1^Liaoning Office for Cancer Prevention and Control, Cancer Hospital of China Medical University, Liaoning Cancer Hospital & Institute, Shenyang, China; ^2^National Cancer Center/National Clinical Research Center for Cancer/Cancer Hospital & Shenzhen Hospital, Chinese Academy of Medical Sciences and Peking Union Medical College, Shenzhen, China

**Keywords:** cancer, healthy literacy, knowledge, China, education

## Abstract

**Background:**

Cancer literacy as a potential health intervention tool directly impacted the success of cancer prevention and treatment initiatives. This study aimed to evaluate the cancer literacy in Northeast China, and explore the factors contributing to urban–rural disparities.

**Methods:**

A cross-sectional survey was conducted in 14 cities across Liaoning Province, China, from August to October 2021, using the multistage probability proportional to size sampling (PPS) method. The survey comprised 4,325 participants aged 15–69 and encompassed 37 core knowledge-based questions spanning five dimensions. Associations between sociodemographic factors and the cancer literacy rate were evaluated using chi-square tests and multivariate logistic regression model.

**Results:**

The overall cancer literacy rate was 66.9% (95% CI: 65.6–68.2%). In the primary indicators, cancer literacy were highest in treatment (75.8, 95% CI: 74.2–77.4%) and early detection (68.2, 95% CI: 66.8–69.6%), followed by basic knowledge (67.2, 95% CI: 65.8–68.6%), recovery (62.6, 95% CI: 60.7–64.5%) and prevention (59.7, 95% CI: 58.2–61.3%). Regarding secondary indicators, the awareness rates regarding cancer-related risk factors (54.7, 95% CI: 52.8–56.5%) and early diagnosis of cancer (54.6, 95% CI: 52.7–56.6%) were notably inadequate. Rural participates exhibited lower cancer literacy across all dimensions compared to urban. Multi-factor analysis showed that factors such as advanced age, limited education or low household income were barriers to health literacy in rural areas.

**Conclusion:**

Strengthening awareness concerning prevention and early detection, particularly among key populations, and bridging the urban–rural cancer literacy gap are imperative steps toward achieving the Healthy China 2030 target.

## Introduction

Cancer stands as a significant public health issue profoundly impacting human health and social progress in the 21st century (1). In recent years, the burden of cancer in China has been on the rise, but it is significantly different between urban and rural areas (2). Factors such as an aging population, economic conditions, and environmental influences have contributed to notably higher incidence and mortality rates of cancer in Northeast China, surpassing the national averages and imposing a heavier disease burden.

In response to these challenges, the Chinese government has implemented a series of cancer control plans and policies. These initiatives include setting phased targets for cancer literacy, aiming for rates of 70% by 2022 and 80% by 2030, respectively (3). Health literacy, defined as an individual’s capability to access, comprehend basic health information and services, and utilize them effectively in fostering personal health decisions, has emerged as a pivotal aspect (4). Numerous studies have highlighted cancer literacy as a fundamental health intervention tool directly impacting the advancement and implementation of cancer prevention and treatment initiatives (5–8). Additionally, cancer literacy strongly correlates with awareness of cancer risks, early diagnosis and standardized treatment levels. Enhanced knowledge about cancer has been associated with reduced cancer risks (9).

However, prevailing research on cancer literacy predominantly focused on singular assessments or specific demographic groups, lacking comprehensive representativeness (10–12). Large-scale, randomized surveys concerning cancer health literacy in China were infrequent and often lack comprehensive scope, hindering an accurate assessment of cancer literacy status. Our study conducted a sampling survey among residents aged 15–69 in Liaoning province across Northeast China. The questionnaires covered basic knowledge, prevention, early detection and intervention, treatment and rehabilitation of cancer. We aimed to evaluate the current level of cancer literacy in Liaoning province. Additionally, we also assessed the differences and related influencing factors between urban and rural areas, providing a reference for future targeted health education and interventions.

## Materials and methods

### Participants

This cross-sectional survey was conducted between August and October 2021 among residents aged 15–69 who had resided in the area for over 6 months within the past year. Approval for this study was obtained from the Ethics Committee of the Cancer Hospital of the Chinese Academy of Medical Sciences (reference number: NCC-007739). Written informed consent was obtained from each participant prior to their inclusion in the study. Liaoning Province, located in the southern part of Northeast China, is a coastal and border province. Encompassing 14 prefecture-level cities, this research ensured comprehensive coverage to accurately evaluate cancer literacy within the province. This study followed the Strengthening the Reporting of Observational Studies in Epidemiology reporting guideline (13).

### Sampling method

Based on the national survey program (14), this study adopted the multistage probability proportional to size sampling (PPS) method, structured across five stages. Initially, one county was randomly selected in each city. Subsequently, within each county, three streets or towns were randomly designated, and within these, two neighborhoods or village committees were further identified. Finally, a random selection of 60 households was made, utilizing the Kish grid method to identify an eligible family member to participate in the survey.

### Survey tool

The tool was designed by the National Cancer Center of China, evaluated by a multi-expert panel, and validated in the national population (14). The Cronbach coefficient was 0.92. The questionnaire was based on three levels of prevention and covers five dimensions (see details in Supplementary Table S1). It comprised true-or-false items (13 questions), single-choice items (13 questions), and multiple-choice items (11 questions). Each correct response was awarded 1 point, while incorrect or unanswered items received no scoring. Additionally, basic information such as age, sex, household registration, marital status, education level, annual income, family history of cancer, and screening history were also collected. Cancer literacy at the population level was calculated as the sum of the final scores of all respondents divided by the number of items to be answered. According to the Healthy China Action (2019–2030), the cancer literacy rate of 70% was defined as “meeting the standard.”

### Quality control

To ensure uniformity and precision throughout the study, all survey personnel underwent comprehensive training. The survey questionnaires were entered into EpiData software (Version 3.1). A meticulous review for any missing data was conducted on the day of the survey. City-level units further performed a reassessment, and if no discrepancies were detected within 10% of randomly selected samples, the questionnaires underwent upload to provincial-level units for meticulous data verification and analysis.

### Statistical methods

Statistical analyses were performed using SPSS software version 23.0. Categorical variables were presented as counts and percentages. Associations between sociodemographic factors and the cancer literacy rate were evaluated using chi-square tests and multivariate logistic regression models. A 2-sided test with a significance level of *α* = 0.05 was employed. Additionally, odds ratios along with their corresponding 95% confidence intervals (CIs) were computed. To enhance accuracy, the cancer literacy estimates were subjected to multistep weighting based on the 2020 Liaoning Province population census data (15). This adjustment primarily accounted for the three key variables of the population distribution of the cities, namely sex (male, female), type of registered permanent residence (urban, rural), and education level (incomplete compulsory education, junior high school, high school, college and above). In addition, the proportions of population distribution in each city and administrative districts among Liaoning province were also considered (Supplementary material).

## Results

### Basic characteristics

In this study, a total of 4,325 participants were recruited for analysis and the response rate was 84.47%. As shown in Table 1, the proportion of the participants in Western Liaoning was the highest (31.6%). The median age of the participants was 51 (41–60) years, and the urban dwellers and females accounted for 64.7 and 57.0%, respectively. The participants with middle levels of education (44.3%) was in higher proportions, which was consistent with the census data (15). Moreover, most participants were Han ethnicity (87.8%) and married (79.5%). Nearly a quarter participants had family history of cancer, and only 18.8% had screening history of cancer.

**Table 1:** The rates of cancer literacy by participant characteristics in Liaoning Province, China, 2021.

Characteristics	Sample (*n*)	%	Cancer literacy, % (95% CI)	*p* value
Administrative divisions^a^				< 0.001
East Liaoning	988	22.8	63.7 (62.2, 65.3)	
South Liaoning	778	18.0	68.4 (65.9, 70.9)	
Western Liaoning	1,368	31.6	65.1 (62.1, 68.2)	
North Liaoning	359	8.3	58.3 (55.3, 61.2)	
Central Liaoning	832	19.2	69.2 (66.9,71.6)	
Type of registered permanent residence				< 0.001
Rural	1,527	35.3	64.0 (60.5, 67.5)	
Urban	2,798	64.7	68.2 (67.0, 69.4)	
Sex				0.835
Male	1,859	43.0	67.0 (65.1, 68.8)	
Female	2,466	57.0	66.8 (64.9, 68.8)	
Ethnicity				0.006
Han ethnic group	3,799	87.8	67.1 (65.7, 68.6)	
Ethnic minority	526	12.2	64.7 (60.6, 68.8)	
Age (years)				< 0.001
15–34	581	13.4	70.7 (68.4, 72.9)	
35–54	1,935	44.7	67.9 (65.5, 70.3)	
55–69	1,809	41.8	64.9 (63.1, 66.8)	
Marital status				< 0.001
Unmarried	361	8.3	68.6 (65.7, 71.5)	
Married	3,439	79.5	65.6 (64.1, 67.1)	
Separated/divorced/widowed	525	12.1	73.7 (69.7, 77.6)	
Education level				< 0.001
Primary school and below	600	13.9	63.2 (59.0, 67.4)	
Middle school	1,914	44.3	65.0 (62.7, 67.3)	
High school	964	22.3	68.7 (66.2, 71.2)	
Associate degree	520	12.0	70.5 (68.6, 72.3)	
Bachelor degree and above	327	7.6	73.4 (70.2, 76.6)	
Occupation				< 0.001
Primary industry	1,349	31.2	62.3 (60.1, 64.5)	
Secondary industry	340	7.9	66.9 (63.2, 70.7)	
Tertiary industry	1,077	24.9	69.4 (66.5, 72.4)	
Retirement or unemployed	1,599	36.0	67.7 (65.5, 70.0)	
Family history of cancer^b^				< 0.001
No	3,316	76.7	66.7 (65.3, 68.1)	
Yes	797	18.4	69.2 (65.4, 73.1)	
Screening history of cancer^b^				< 0.001
No	3,393	78.5	66.3 (64.7, 67.9)	
Yes	811	18.8	70.8 (68.4, 73.1)	
Self-reported health status				< 0.001
Good or relatively good	3,037	70.2	67.1 (65.5, 68.7)	
Average	1,128	26.1	65.6 (63.7, 67.6)	
Poor or relatively poor	160	3.7	72.2 (60.3, 84.0)	
Annual household income *per capita* (CNY)^c^				< 0.001
< 10,000	1,025	23.7	62.8 (60.5, 65.2)	
10,000 ~ < 16,667	1,072	24.8	66.4 (63.0, 69.8)	
16,667 ~ < 30,000	1,131	26.2	68.5 (66.5, 70.6)	
≥ 30,000	1,097	25.4	68.0 (65.5, 70.5)	

CNY, Chinese Yuan.

a The cities included in each administrative division were determined following the government document of Liaoning: (1) East Liaoning: Dandong, Fushun, Benxi; (2) South Liaoning: Dalian, Yingkou; (3) West Liaoning: Chaoyang, Jinzhou, Huludao, Fuxin, Panjin; (4) North Liaoning: Tieling; (5) Central Liaoning: Shenyang, Anshan, Liaoyang.

b The date was missing.

c The income was classified according to quartiles.

### The overall rate of cancer literacy

After multiple adjustments, the overall cancer literacy rate was 66.9% (95% CI: 65.6–68.2%) in Liaoning Province, China. There were significant differences among the various administrative regions (Table 1). Overall, the rate was highest in Central region (69.2, 95% CI: 66.9–71.6%), followed by South (68.4, 95% CI: 65.9–70.9%), and the lowest was in North (58.3, 95% CI: 55.3–61.2%).

### The overall rates of cancer literacy by participant characteristics

**Figure 1:**
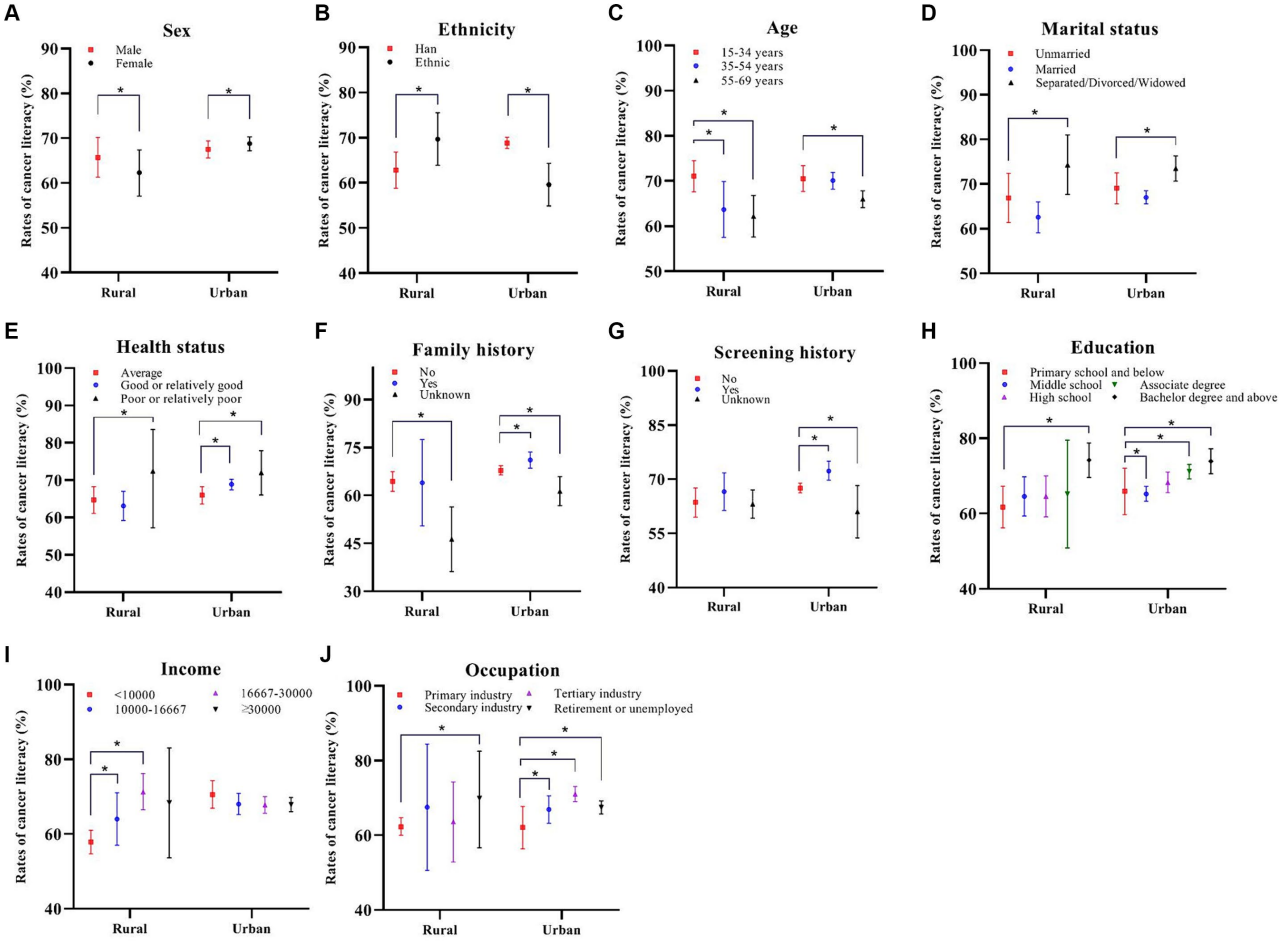
Comparison of the rates of cancer literacy between different areas and sex **(A)**, ethnicity **(B)**, age **(C)**, marital status **(D)**, health status **(E)**, family history **(F)**, screening history **(G)**, education **(H)**, income **(I)**, occupation **(J)**. ^*^p < 0.01.

As shown in Table 1, there was a significant difference in overall awareness rates between urban and rural residents, with urban residents having a higher rate (68.2% VS 64.0% in rural areas). Additionally, Han Chinese (VS ethnic minorities, 67.1% VS 64.7%), participates aged 15–34 years (VS 55–69 years, 70.7% VS 64.9%), and those in the tertiary sector (VS primary sector, 69.7% VS 62.3%) had higher overall awareness rates. Notably, higher educational levels correlated positively with increased awareness rates (*p* < 0.001). Participants with a family history of cancer (69.2% VS 66.7% without family history) and those with a screening history (70.8% VS 66.3% without screening history) had higher awareness rates (*p* < 0.001), and the trend were more pronounced in urban areas (Figure 1). With increasing income, awareness rates were also rise, particularly in rural areas (*p* < 0.01). Among all participates, there was no significant variation in overall awareness rates between different genders (*p* = 0.835). However, in urban areas, females exhibited higher awareness rates compared to males, whereas the opposite trend was observed in rural areas (*p* < 0.01) (Figure 1).

Multivariate analysis revealed that education level emerged as the pivotal factor influencing whether the cancer literacy rate met the standard (Table 2). In addition, in rural areas, it was more difficult for participates aged 55–69 years old to meet the cancer awareness standard than those aged 15–34 years old. Conversely, participates with annual household income *per capita* over 10,000 were more likely to meet the standard. Notably, in urban areas, participates with a family history of cancer and a history of cancer screening were more likely to meet the cancer awareness standard.

**Table 2:** Multivariate analysis of the overall rates of cancer literacy between urban and rural areas in Liaoning Province, China, 2021.

Variables	Rural		Urban	
	OR (95% CI)	*p* value	OR (95% CI)	*p* value
Age (years)				
15–34	1 (Reference)		1 (Reference)	
35–54	0.62 (0.42, 0.91)	0.014	1.13 (0.84, 1.51)	0.413
55–69	0.46 (0.30, 0.70)	< 0.001	0.84 (0.61, 1.16)	0.299
Education level				
Primary school and below	1 (Reference)		1 (Reference)	
Middle school	1.35 (1.03, 1.78)	0.029	1.29 (0.87, 1.90)	0.207
High school	1.98 (1.28, 3.06)	0.002	1.79 (1.19, 2.67)	0.005
Associate degree	2.28 (1.26, 4.13)	0.006	1.67 (1.08, 2.60)	0.022
Bachelor degree and above	2.02 (0.80, 5.11)	0.136	2.79 (1.72, 4.53)	< 0.001
Occupation				
Tertiary industry	1 (Reference)		1 (Reference)	
Primary industry	0.83 (0.57, 1.21)	0.336	0.41 (0.28, 0.60)	< 0.001
Secondary industry	0.42 (0.19, 0.95)	0.037	0.85 (0.63, 1.14)	0.274
Retirement or unemployed	0.96 (0.58, 1.58)	0.861	1.07 (0.86, 1.33)	0.537
Family history of cancer				
No	1 (Reference)		1 (Reference)	
Yes	1.03 (0.76, 1.41)	0.836	1.40 (1.14, 1.73)	0.002
Screening history of cancer				
No	1 (Reference)		1 (Reference)	
Yes	0.97 (0.71, 1.32)	0.830	1.57 (1.26, 1.95)	< 0.001
Annual household income *per capita* (CNY)				
< 10,000	1 (Reference)		1 (Reference)	
10,000 ~ < 16,667	1.54 (1.19, 2.01)	0.001	0.90 (0.67, 1.21)	0.476
16,667 ~ < 30,000	1.61 (1.16, 2.23)	0.005	0.79 (0.59, 1.05)	0.107
≥ 30,000	1.23 (0.78, 1.93)	0.366	0.75 (0.56, 1.02)	0.065

CNY, Chinese Yuan.

### The rates of cancer literacy in various dimensions

As shown in Table 3, among the five primary indicators, the highest awareness was in cancer treatment at 75.8% (95% CI: 74.2–77.4%) while the lowest was in cancer prevention at 59.7% (95% CI: 58.2–61.3%). The awareness rates for the basic sense of cancer, early diagnosis and treatment, and patient recovery were 67.2% (95% CI: 65.8–68.6%), 68.2% (95% CI: 66.8–69.6%), and 62.6% (95% CI: 60.7–64.5%), respectively.

**Table 3:** The rates of primary indicators of cancer literacy in Liaoning Province, China, 2021.

Indicators	Cancer literacy, % (95% CI)		
	Overall	Rural	Urban
1. Basic sense of cancer	67.2 (65.8, 68.6)	65.0 (61.5, 68.4)	68.2 (66.9, 69.5)
2. Cancer prevention	59.7 (58.2, 61.3)	55.7 (51.8, 59.7)	61.5 (60.0, 62.9)
3. Early detection and intervention	68.2 (66.8, 69.6)	65.5 (62.0, 69.0)	69.4 (68.0, 70.7)
4. Cancer treatment	75.8 (74.2, 77.4)	73.3 (69.2, 77.4)	76.9 (75.4, 78.3)
5. Patients recovery	62.6 (60.7, 64.5)	58.9 (54.1, 63.7)	64.2 (62.3, 66.0)

In secondary indicators (Table 4), the rates were relatively high for the main treatment of cancer (86.9, 95% CI: 84.8–88.9%), the significance of early detection (76.4, 95% CI: 74.7–78.1%), and regular check (75.8, 95% CI: 73.5–78.0%). However, the awareness rate in the early diagnosis of cancer was the lowest (54.6, 95% CI: 52.7–56.6%). The awareness rate of cancer-related risk factors was also relatively low, such as the tertiary indicators concerning the unhealthy life style factors (35.0, 95% CI: 32.4–37.6%) and the infection factors (38.1, 95% CI: 35.5–40.7%) (Supplementary Table S3). Except for the secondary indicator concerning the significance of early detection, urban residents had higher awareness rates across all indicators compared to rural residents.

**Table 4:** The rates of secondary indicators of cancer literacy in Liaoning Province, China, 2021.

Indicators	Cancer literacy, % (95% CI)		
	Overall	Rural	Urban
4.3. Main treatment of cancer	86.9 (84.8, 88.9)	83.2 (77.8, 88.6)	88.4 (86.8, 90.1)
3.1. Significance of early detection	76.4 (74.7, 78.1)	76.8 (72.7, 81.0)	76.2 (74.5, 77.9)
4.2. Regular check	75.8 (73.5, 78.0)	72.4 (66.7, 78.1)	77.2 (75.2, 79.2)
3.4. Early treatment of cancer	75.6 (73.7, 77.4)	73.4 (69.2, 77.6)	76.5 (74.6, 78.4)
4.1. Standardized treatment	72.1 (70.5, 73.7)	70.6 (66.9, 74.2)	72.7 (71.0, 74.5)
1.2. Basic knowledge of cancer	71.9 (70.5, 73.4)	69.3 (65.8, 72.9)	73.1 (71.6, 74.5)
3.2. Identification of warning symptoms	70.6 (68.9, 72.3)	66.5 (62.0, 71.0)	72.4 (70.9, 73.9)
5.2. Psychological rehabilitation	70.0 (67.4, 72.6)	66.2 (60.1, 72.4)	71.6 (69.1, 74.2)
2.2. Prevention measures	64.8 (63.1, 66.5)	62.3 (58.1, 66.6)	65.9 (64.3, 67.5)
1.1. Attitudes and beliefs	63.4 (61.8, 65.0)	61.5 (57.4, 65.6)	64.3 (62.8, 65.7)
5.1. Physiological rehabilitation	58.9 (56.6, 61.2)	55.2 (49.7, 60.8)	60.5 (58.2, 62.7)
2.1. Risk factors	54.7 (52.8, 56.5)	49.1 (44.2, 54.0)	57.0 (55.4, 58.7)
3.3. Early diagnosis of cancer	54.6 (52.7, 56.6)	51.2 (46.6, 55.8)	56.1 (54.1, 58.1)

## Discussion

Understanding the level of cancer literacy within the population is crucial for effectively implementing tailored cancer health education initiatives. (16). This study revealed a significant disparity in the dissemination of cancer knowledge between urban and rural areas, shedding light on the blind spots in Northeast China. Employing a validated and reliable survey tool, the study adopted a randomized sampling approach for face-to-face interviews. After multi-step weighted adjustment, the overall rate of cancer literacy was 66.2%. Although it did not reach the target set in China (70%), this rate surpassed figures from South China (63.2%), Southwest China (59.0%), and Northwest China (57.1%) (14). Similarly, in Liaoning Province, the awareness rate was also closely related to geographical location. The Central Liaoning region had the highest awareness rate, followed by Southern Liaoning. This may be attributed to Central and Southern Liaoning’s status as economic and coastal centers, respectively, boasting concentrated medical institutions and professionals. Increased allocation of health resources positively impact the dissemination of health knowledge (17). Consequently, targeted strategies in regions with relatively low cancer literacy should focus not only on health education but also on bolstering medical resources and policies to foster equitable development of the medical landscape.

In China, the rural population exhibit suboptimal comprehension of cancer, a factor closely linked to increased cancer susceptibility and reduced survival rates (2). In this study, disparities in education level, age and income emerged as crucial factors influencing awareness rates. In rural areas, the majority of older people possess lower education levels, and their children frequently reside elsewhere, limiting their access to vital information. Additionally, people with lower incomes, grappling with greater financial demands, may have limited time to focus on additional information, resulting in reduced attention toward personal health concerns (18). In addition to demographic factors, limited health educational resources and delayed information dissemination hinder timely access to the latest cancer knowledge in rural areas. Moreover, the rural population tends to delayed treatment and misunderstanding due to fear, embarrassment and traditional beliefs (19, 20). Notably, participates with a family history of cancer and experience with cancer screenings exhibited relatively higher health literacy, consistent with previous research (21). People with higher economic incomes and access to ample medical resources are better positioned to prioritize their health and access high-quality medical services. This indirectly emphasizes that knowledge about cancer can significantly influence individuals’ health attitudes and proactive health actions (5, 22, 23).

Targeted health education is an effective way to improve public health literacy and reduce the disease burden. This study conducted a comprehensive evaluation of cancer literacy across five dimensions, uncovering the lowest awareness rate in cancer prevention, particularly regarding knowledge of cancer risk factors such as unhealthy lifestyles and infections. A nationwide population-based survey in Japan revealed low awareness regarding the risk of unhealthy lifestyles for cancer such as alcohol consumption and obesity, but high awareness regarding the risk of viral infections such as HPV (24). Similarly, Australian adults also exhibited limited awareness of dietary risk factors (25). Notably, one-third of cancers are preventable, and awareness of cancer-causing factors directly impacts the implementation and promotion of primary cancer prevention (26). In China, approximately 1.036 million cancer-related deaths occur annually due to 23 carcinogenic factors, with modifiable risk elements such as unhealthy lifestyles accounting for over 40% of cancer incidence and mortality (27). Furthermore, China also has a high prevalence of biological infections like HBV and HPV (28, 29). Consequently, enhancing the awareness of carcinogenic factors and promoting healthy lifestyles stands as a cost-effective method for cancer prevention.

In secondary prevention, recognizing personal cancer risk signs and undergoing targeted checks stand as pivotal steps toward early cancer detection. Within this survey, participates exhibited an awareness rate of over 70% regarding recognizing early signs and symptoms of cancer, attributed partly to the decade-long healthy education initiatives by Liaoning Province’s government in cancer symptom and risk factor awareness. However, this was still behind other high-income countries. For instance, studies in Malaysia revealed that 86.6% of participants identified “blood in stool” as a sign of colorectal cancer (30), while 89% recognized “worsening or changing cough” as a symptom of lung cancer (21). Regrettably, this study observed limited awareness regarding when to undergo screenings and which targeted checks to pursue, with an awareness rate of only 54.6%. This might be due to the low coverage of current population screening programs and restricted access to medical resources. On the other hand, this lack of risk awareness also affected their motivation to get help, and thus delayed screening behavior (31, 32). To some extent, this also resulted in the low screening participation rate in Liaoning Province (33). Investigations into why people were reluctant to undergo cancer screening revealed that 50% were due to ignorance about cancer (34). This diminished compliance directly affects the proportion of early cancer diagnoses, consequently reducing survival rates (35). In China, some measures are required to correctly comprehend cancer screenings to reduce cancer mortality rates and enhance survival outcomes through early detection, diagnosis, and treatment (36–38).

For cancer prevention and control, nations worldwide have been taking proactive steps. Several countries have integrated the HPV vaccine into immunization schedules and incorporated cancer screening into health insurance, requiring residents to undergo regular screenings (39–41). In the future, we should promote the healthy lifestyles, particularly through widespread cancer knowledge dissemination in schools, enterprises and villages. Secondly, many cities should be encouraged to subsidize vaccines and cancer screening projects to enhance public awareness. Notably, policy formulation should consider rural realities, optimizing medical resource allocation and focusing on disseminating knowledge about cancer risk factors and early screening, thereby improving the accessibility and interest of health materials.

### Limitations

This study also had several limitations. Despite employing multi-step adjustments to enhance the representativeness of the sample size, there was still some sampling error, and the reported cancer literacy in this study might be overestimated or underestimated. Additionally, certain participants, especially in rural areas, with limited education, might have misunderstood the questions, leading to a partial underestimation of the awareness rate.

## Conclusion

This study represents the first cross-sectional survey of cancer literacy in Northeast China. The results revealed that in Liaoning Province, the overall cancer literacy stood at 66.9%, with insufficient knowledge regarding cancer risk factor recognition and early diagnosis. Particularly noteworthy was the significantly lower cancer literacy in rural areas compared to urban areas, with distinct influencing factors. It is imperative for the government to fully recognize the importance and urgency of enhancing cancer literacy through the development of targeted health education programs. These programs should be tailored specifically for the rural population, characterized by the advanced age, limited education, and low-income levels, in order to achieve phased targets.

## Data availability statement

The raw data supporting the conclusions of this article will be made available by the authors, without undue reservation.

## Ethics statement

Approval for this study was obtained from the Ethics Committee of the Cancer Hospital of the Chinese Academy of Medical Sciences (reference number: NCC-007739). Written informed consent was obtained from each participant prior to their inclusion in the study.

## Author contributions

ML: Conceptualization, Data curation, Formal analysis, Methodology, Software, Writing – original draft, Writing – review & editing. PN: Writing – review & editing, Data curation. TZ: Methodology, Writing – review & editing, Formal analysis. YL: Project administration, Writing – review & editing, Data curation. BZ: Conceptualization, Writing – review & editing.
