# Supplementary material for: Cancer literacy differences of basic knowledge, prevention, early detection, treatment and recovery: a cross-sectional study of urban and rural residents in Northeast China
Source: Front Public Health. 2024 May 14;12:1367947. doi: 10.3389/fpubh.2024.1367947 (PMC11130368; doi:10.3389/fpubh.2024.1367947)
Supplement: Supplementary file 1 [file Table_1.DOCX]

**Table S1. The survey on the rates of cancer literacy in Liaoning Province, China, 2021**

| **Types** | **Problems** |
| --- | --- |
| **Section 1: True or false questions**  **Directions: In this section, there are 13 questions. You should decide on the only one correct choice and fill in the brackets with T (for TRUE) or F (for FALSE). If you have no idea, please fill in the brackets with D (for Don't know).** | **A01** Cancer usually takes many decades to develop. (√) |
|  | **A02** By taking comprehensive measures of cancer prevention and control, some areas of China have seen a decreasing incidence and deaths of cancer. (√) |
|  | **A03** In addition to surgical treatment, cancer treatment include radiotherapy, chemotherapy, targeted therapy, immune therapy, endocrine therapy, as well as the combination of traditional Chinese and Western medicine, etc. (√) |
|  | **A04** Patients only need to actively strengthen the natural immunity during the rehabilitation of cancer, because psychological rehabilitation cannot help to maintain the stability of patient’s disease. (ⅹ) |
|  | **A05** Vaccination (such as HPV vaccine) can prevent some cancers. (√) |
|  | **A06** We can directly use the treatment which showed good curative effects on others, regardless of the pathological subtype and stage of cancer. (ⅹ) |
|  | **A07** Some cancers (such as breast cancer, colon cancer, etc.) have certain heritability, and the individuals with family of cancer should pay more attention to their health. (√) |
|  | **A08** Cancer screening is a special physical examination for cancer based on the actual situation of individuals, which has been able to detect some common types of cancers at an early stage. (√) |
|  | **A09** If someone has a persistent dry cough, finds blood in sputum, or has other symptoms, he/she should go to the hospital for further examination of lung cancer (√) |
|  | **A10** If the mole on your body becomes darker and larger in a short time, you can excise it by yourself or chose to wait for further development, while don’t have to go to the hospital immediately. (ⅹ) |
|  | **A11** Many cancers can be cured by folk prescription, taking health care products, or following the treatment plan in the advertisement. (ⅹ) |
|  | **A12** Cancer is not an incurable disease, and some cancer patients are able to survival for decades. (√) |
|  | **A13** The most common first symptom of esophageal cancer is difficulty swallowing or pain. (√) |
| **Section 2: Single choice question**  **Directions: In this section, there are 13 questions. For each of them there are four choices marked A, B, C and D. You should decide on the only one correct choice and mark the corresponding letter with “√”. If you have no idea, please choose D.** | **B01 Which of the following statement is true about the relationship between cancer and lifestyle? C**  A Lifestyle determines the risk of cancer, so individuals who are heavy smoker or alcoholics would be diagnosed with cancer sooner or later.  B Lifestyle has no relationship with cancer.  C Lifestyle is closely associated with cancer, and an unhealthy lifestyle increases the risk of cancer.  D Don’t know. |
|  | **B02 Which is the most common cancer in China currently? A**  A Lung cancer.  B Bladder cancer.  C Pancreatic cancer.  D Don’t know. |
|  | **B03 Which of the following statement is true about the trend of cancer incidence in China in recent years? A**  A The incidences of lung cancer, breast cancer and colorectal cancer are increasing.  B The incidences of lung cancer, breast cancer and colorectal cancer are decreasing.  C The incidences of lung cancer, breast cancer and colorectal cancer remain constant.  D Don’t know. |
|  | **B04 Which of the following statement is true about the prevention of cancer? B**  A The occurrence of cancer is only related to a person’s health status, regardless of his/her age.  B Cancer could be occurred at any age, so we should develop a healthy lifestyle from an early age.  C Middle-aged and elderly people show high cancer incidence, so there is no need for teenagers to worry about getting cancer.  D Don’t know. |
|  | **B05 Which of the following statement is true about cancer? C**  A It’s mainly related to genetic factors.  B It’s mainly related to lifestyle.  C It’s related to both individual factors and environmental factors.  D Don’t know. |
|  | **B06 Which of the following statement is true about cancer? C**  A All types of cancer are contagious so we should keep away from cancer patients.  B Some types of cancer are contagious. For example, there are whole families who get cancer.  C Cancer itself is not contagious, but bacteria and virus relating to cancer are contagious.  D Don’t know. |
|  | **B07 Which of the following statement is true about early detection of cancers? A**  A You should choose professional medical institutions and appropriate physical examination.  B You can choose any physical examination according to your own ideas and interests.  C There is no need to take cancer examinations if you have done a general physical examination.  D Don’t know. |
|  | **B08 Which of the following statement is true about the frequency of taking cancer examinations? B**  A A one-off cancer examination is enough because cancer develops slowly.  B The interval period of cancer examinations is dependent on your age and previous diagnosis of your physical examinations.  C It’s unnecessary to take cancer examinations unless you do not feel well.  D Don’t know. |
|  | **B09 If frequent toileting occurs recently, and the stool gets bloody and thin , you should go to the hospital to further exam for: B**  A liver cancer.  B Colorectal cancer.  C Pancreatic cancer.  D Don’t know. |
|  | **B10 Which of the following statement is true about the survival of cancer? A**  A The earlier cancer is detected, the better the therapeutic efficacy is, and the longer the survival time is.  B The survival time is related to the malignant degree of the tumor, but not to the time of diagnosis.  C As long as someone is diagnosed with cancer, his/her survival time will not be too long even diagnosed at an early stage.  D Don’t know. |
|  | **B11 Which of the following statement is true about cancer examinations? B**  A It must be cancer as long as the indicators show an abnormal value during cancer examinations.  B Further diagnosis and treatment are needed if the indicators show an abnormal value during cancer examinations.  C There is no need to concern about abnormal indicators if you feel well.  D Don’t know. |
|  | **B12 Which of the following statement is true about anticancer drugs? C**  A As long as you take it, the therapeutic efficacy remains constant. You can crush it yourself or mix it with other foods.  B If you forget to take or take fewer tablets of anti-cancer drugs at one time, you should increase your dosage at the next time .  C Taking medicine should follow the doctor's advice because the therapeutic efficacy will be different when taken before and after meals.  D Don’t know. |
|  | **B13 Which of the following statement is true about reexamination? C**  A The more frequent and more comprehensive the reexamination is, the better the prognosis will be.  B As long as you take medicine on time, you don't need to go to the hospital for further consultation to avoid increasing the medical burden  C Reexamination regularly is necessary so as to detect and treat metastatic tumor and reoccurrence timely.  D Don’t know. |
| **Section 3: Multiple choice question**  **Directions: In this section, there are 11 questions. In this section, each question has two or more correct answers, you should decide on the correct choices and mark the corresponding letters with “√”. If you have no idea, please select E.** | **C01 What of the followings are associated with the cancer? ABCD**  A Chemical factor, such as toxic organic substances.  B Physical factors, such as radiation.  C Psychological factors, such as excessive stress and mental tension.  D Behavioral factors, such as an unhealthy lifestyle.  E Don’t know. |
|  | **C02 Which of the following biological factors can increase the risk of cancer? BCD**  A Probiotics in the gut can increase the risk of bowel cancer.  B Infection with Helicobacter pylori can increase the risk of stomach cancer.  C Infection with hepatitis B virus (HBV) can increase the risk of liver cancer.  D Infection with human papillomavirus (HPV) can increase the risk of cervical cancer.  E Don’t know. |
|  | **C03 Which of the following unhealthy lifestyle habits can increase the risk of cancer? ABD**  A Lack of exercise.  B Smoking, drinking.  C Excessive attention to personal hygiene.  D Irregular diet habits.  E Don’t know. |
|  | **C04 To prevent the development of cancer, you should do: ABC**  A Keep yourself in a good mood.  B Moderate exercise.  C Quit smoking and limit alcohol intake.  D Arrange meals according to your personal preference.  E Don’t know. |
|  | **C05 Which of the following measures can effectively reduce the incidence and mortality of cancer? ABCD**  A regular cancer examinations.  B Participate in cancer screening.  C Receive health education, and improve health literacy.  D Treat chronic infections actively (such as chronic hepatitis B).  E Don’t know. |
|  | **C06 Which of the following statements is correct about cancer prevention and treatment: ABD**  A Some cancers are completely preventable by taking active measures.  B Some cancers can be cured with the current level of medical care.  C Cancer is developing so fast that cannot be cured even if it is detected early.  D Some cancers are incurable but can be treated to relieve suffering and prolong life.  E Don’t know. |
|  | **C07 Which of the following statements is true about cancer screening: ABCD**  A Early gastrointestinal cancer can be detected by endoscopy.  B Early lung cancer can be detected by LDCT of the chest.  C Early liver cancer can be detected by abdominal B-mode ultrasound combined with alpha-fetoprotein examination.  D Early breast cancer can be detected by breast ultrasound combined with mammography.  E Don’t know. |
|  | **C08 Which of the following symptoms may be a warning sign of cancer and should be noted: ABCD**  A Persistent hoarseness, and dry cough.  B Apparent weight loss for unknown reasons.  C Recurrent fever and weakness of unknown reasons.  D Unexplained bleeding such as blood in urine and stool.  E Don’t know. |
|  | **C09 Which of the following groups of people are at high risk of cancer? ABCD**  A Obese people.  B People aged 50 and over.  C People who are introverted and sulky.  D People who have a first-degree relative diagnosed with cancer.  E Don’t know. |
|  | **C10 Which of the following statement is correct about pain in cancer patients: AB**  A Cancer pain is one of the common symptoms of cancer patients, but most can be relieved by treatment.  B If there is cancer pain, you should follow the doctor's recommendations to relieve the pain.  C Pain occurs whenever there is cancer and there is no way to relieve it.  D Once a cancer patient has pain, it means that he/she is in the advanced stages.  E Don’t know. |
|  | **C11 For a better recovery, cancer patients should do: ABC**  A Take drugs reasonably, deal with pain and control the disease actively.  B Have a balanced diet and receive nutritional support if necessary.  C Regular reexamination, timely detection of lesions and receive interventions.  D Stay in bed for long periods of time, conserve energy, and refuse any exercise.  E Don’t know. |
| **Section 4:** **Basic information** | **D01.** **Gender:** ① male ② female |
|  | **D02. Date of birth:** ________ |
|  | **D03.** **Your** **ethnicity:**  ①The Han ② The Zhuang ③ The Hui ④ The Manchu ⑤ The Uygur ⑥ The Miao ⑦ The Yi ⑧ The Tujia ⑨ Other  **D04. Your marital status is:**  ①Unmarried ② Married ③ Separated ④ Divorced ⑤ Widowed |
|  | **D05. Your educational level:**  ① Never received formal education  ② Graduated from primary school  ③ Graduated from junior high school  ④ Graduated from senior high School / technical secondary school / technical school  ⑤ Graduated from college  ⑥ Bachelor degree  ⑦ Master degree and above |
|  | **D06. Your occupation is:**  ① Agricultural personnel (agriculture, forestry, animal husbandry and fisheries)  ② Professional and technical personnel (teachers, lawyers, engineers, etc.)  ③ Civil servants and staff of public institutions  ④ Staff of factory and mining enterprises (mining, manufacturing, construction workers, etc.)  ⑤ Commercial retail or service personnel (wholesale and retail merchants, catering workers, etc.)  ⑥ School students  ⑦ Active military personnel  ⑧ Not employed  ⑨ Retired persons  ⑩ Medical workers (doctors, nurses, public health workers, etc.) |
|  | **D07.** **Numbers of people in your household: _____** |
|  | **D08.** **Over the past year, your annual household income was approximately ___ CNY** |
|  | **D09. Does anyone in your family (your grandparents, parents and siblings) suffer from cancer?**  ① Yes ② No ③ I don't know |
|  | **D10. Do you smoke?**  ① Yes, smoke daily. Average daily numbers of cigarettes_____ (please convert the number of packs)  ② Yes, but not every day. Average weekly numbers of cigarettes_____ (please convert the number of packs)  ③ I used to smoke, but now I quit smoking.  ④ Never smoked. |
|  | **D11. Have you ever been screened for cancer (within five years)?**  ① Yes ② No ③ I don't know |
|  | **D12. Your height is____ centimeter, your weight is____ kilogram.** |
|  | **D13. Your type of household registration is:**  ① Agricultural household registration ② Non-agricultural household registration |
|  | **D14. In the past year, you consider your health to be:**  ① Good ② Relatively good ③ In general ④ Relatively bad ⑤ Bad |

**Table S2. The indicators and corresponding items at five dimensions**

| **Primary indicators** | **Secondary indicators** | **Tertiary indicators** | **Item** |
| --- | --- | --- | --- |
| 1 Basic sense of cancer | 1.1Attitudes and beliefs | 1.1.1 Cancer is a chronic disease | A01 |
|  |  | 1.1.2 Cancer is preventable and treatable | C06 |
|  |  | 1.1.3 Cancer is closely related to life styles | B01 |
|  |  | 1.1.4 Cancer is not contagious | B06 |
|  |  | 1.1.5 Humans can coexist with cancer | A12 |
|  | 1.2Basic knowledge | 1.2.1 Epidemiological knowledge | A02 |
|  |  |  | B02 |
|  |  |  | B03 |
|  |  | 1.2.2 Etiological knowledge | C01 |
| 2 Cancer prevention | 2.1 Risk factors | 2.1.1Family history of cancer | A07 |
|  |  | 2.1.2Unhealthy life style | C03 |
|  |  | 2.1.3Environment | B05 |
|  |  | 2.1.4Infection | C02 |
|  | 2.2Prevention measures | 2.2.1Vaccine | A05 |
|  |  | 2.2.2Healthy life style | B04 |
|  |  |  | C04 |
|  |  | 2.2.3Others | C05 |
| 3 Early detection and intervention | 3.1Significance of early detection | 3.1.1Benefits of regular physical examination | A08 |
|  |  | 3.1.2Benefits of early detection and intervention | B10 |
|  | 3.2 Identification of warning symptoms | 3.2.1Warning signs of respiratory cancer | A09 |
|  |  | 3.2.2Warning signs of digestive system cancer | A13 |
|  |  |  | B09 |
|  |  | 3.2.3Warning signs of other cancer | C08 |
|  | 3.3 Early diagnosis of cancer | 3.3.1Screening population | C09 |
|  |  | 3.3.2Screening method | B07 |
|  |  |  | C07 |
|  | 3.4 Early treatment of cancer | 3.4.1Receive treatment timely | A10 |
|  |  |  | B11 |
| 4 Cancer treatment | 4.1 Standardized treatment | 4.1.1Doctor treatment | A06 |
|  |  |  | B12 |
|  |  | 4.1.2Others | A11 |
|  | 4.2Regular check | 4.2.1Take regular check | B08 |
|  |  |  | B13 |
|  | 4.3Main treatment of cancer | 4.3.1Non operative therapy | A03 |
| 5 Patients recovery | 5.1 Physiological rehabilitation | 5.1.1Rehabilitation method | C11 |
|  |  | 5.1.2Cancer pain management | C10 |
|  | 5.2Psychological rehabilitation | 5.2.1Positive attitude | A04 |
