# Supplementary material for: Cancer literacy differences of basic knowledge, prevention, early detection, treatment and recovery: a cross-sectional study of urban and rural residents in Northeast China
Source: Front Public Health. 2024 May 14;12:1367947. doi: 10.3389/fpubh.2024.1367947 (PMC11130368; doi:10.3389/fpubh.2024.1367947)
Supplement: Supplementary file 2 [file Table_2.DOCX]

**Data weighting and calculation:**

The aim of this process was to adjust the sample proportion of the main variables to match Statistics on the 2020 Liaoning Population Census.

Both the sample and census population were first classified into 16 layers by sex (male, female), type of registered permanent residence (urban, rural), and education level (incomplete compulsory education, junior high school, high school, college and above), and then the proportions of each layer for every city were adjusted, during which weight 1 was developed.

Similarly, the population distributions of each administrative districts were adjusted by weight 2 due to the discrepant compliance among cities with the sampling protocol (the other one of the non-representativeness weights). It was assumed that the weighted data were basically representative of each administrative division after the former two steps, and hence, the awareness rate could be regarded as a weighted mean.

Weight 3 referred to the proportions of population in each administrative division among Liaoning province. The coefficients applied in calculations of cancer literacy varied for the city, district and provincial levels, which were weight 1, (weight 1* weight 2), and (weight 1* weight 2 *weight 3), respectively.

Notes: The cities included in each administrative division were determined following the government document of Liaoning: (1) East Liaoning: Dandong, Fushun, Benxi; (2) South Liaoning: Dalian, Yingkou; (3) West Liaoning: Chaoyang, Jinzhou, Huludao, Fuxin, Panjin; (4) North Liaoning: Tieling; (5) Central Liaoning: Shenyang, Anshan, Liaoyang.
