# Supplementary material for: Cancer literacy differences of basic knowledge, prevention, early detection, treatment and recovery: a cross-sectional study of urban and rural residents in Northeast China
Source: Front Public Health. 2024 May 14;12:1367947. doi: 10.3389/fpubh.2024.1367947 (PMC11130368; doi:10.3389/fpubh.2024.1367947)
Supplement: Supplementary file 3 [file Table_3.docx]

| **Indicators** | **Cancer literacy, % (95% CI)** | | |
| --- | --- | --- | --- |
|  | **Overall** | **Rural** | **Urban** |
| 4.3.1 Non operative therapy treatment of cancer | 86.9 (84.8, 88.9) | 83.2 (77.8, 88.6) | 88.4 (86.8, 90.1) |
| 3.1.1 Benefits of regular physical examination | 84.4 (82.4, 86.4) | 81.4 (76.5, 86.3) | 85.7 (83.8, 87.6) |
| 4.1.2 Other standardized treatment of cancer | 79.4 (77.3, 81.5) | 79.6 (75.4, 83.9) | 79.3 (77.0, 81.7) |
| 3.2.2 Warning signs of digestive system cancer | 79.3 (77.3, 81.3) | 74.0 (68.9, 79.2) | 81.6 (79.9, 83.2) |
| 1.2.1 Cancer epidemiology knowledge | 76.7 (75.2, 78.2) | 75.3 (71.8, 78.8) | 77.4 (75.9, 78.9) |
| 2.1.1 Risk factors (family history of cancer) | 76.7 (75.2, 78.2) | 75.8 (70.3, 81.3) | 77.4 (75.9, 78.9) |
| 4.2.1 Take regular check | 75.8 (73.5, 78.0) | 72.4 (66.7, 78.1) | 77.2 (75.2, 79.2) |
| 3.4.1 Receive treatment timely | 75.6 (73.7, 77.4) | 73.4 (69.2, 77.6) | 76.5 (74.6, 78.4) |
| 1.1.3 Cancer is closely related to life styles | 74.0 (71.8, 76.3) | 72.3 (67.2, 77.5) | 74.8 (72.4, 77.1) |
| 1.1.4 Cancer is not contagious | 72.2 (69.9, 74.6) | 69.5 (64.0, 75.1) | 73.4 (71.1, 75.8) |
| 3.2.1 Warning signs of respiratory cancer | 72.0 (69.4, 74.6) | 68.7 (62.3, 75.2) | 73.5 (71.1, 75.9) |
| 3.2.3 Warning signs of other cancer | 70.6 (68.9, 72.3) | 66.5 (62.0, 71.0) | 72.4 (70.9, 73.9) |
| 5.2.1 The positive attitude of psychological rehabilitation | 70.0 (67.4, 72.6) | 66.2 (60.1, 72.4) | 71.6 (69.1, 74.2) |
| 2.2.1 Prevention measures (Vaccine) | 68.7 (66.3, 71.1) | 67.5 (61.9, 73.1) | 69.3 (66.7, 72.7) |
| 3.1.2 Benefits of early detection and intervention | 68.4 (66.0, 70.8) | 66.7 (64.2, 69.2) | 72.3 (66.9, 77.7) |
| 4.1.1 Doctor standardized treatment | 68.4 (66.5, 70.4) | 66.0 (61.5, 70.6) | 69.5 (67.5, 71.4) |
| 2.2.2 Prevention measures (Healthy life style) | 67.3 (65.4, 69.3) | 64.7 (60.2, 69.3) | 68.5 (66.5, 70.4) |
| 2.1.3 Risk factors (Environment) | 66.2 (63.4, 68.9) | 56.9 (50.4, 63.4) | 70.2 (67.7, 72.7) |
| 5.1.1 The method of physiological rehabilitation | 64.2 (61.6, 66.7) | 60.4 (54.4, 66.3) | 65.8 (63.2, 68.5) |
| 1.1.1 Cancer is a chronic disease | 63.9 (61.4, 66.5) | 64.2 (58.4, 70.1) | 63.8 (61.3, 66.4) |
| 1.1.5 Humans can coexist with cancer | 63.9 (61.4, 66.3) | 61.6 (55.9, 67.3) | 64.9 (62.2, 67.5) |
| 3.3.2 Screening method | 63.4 (61.4, 65.4) | 60.5 (55.8, 65.2) | 64.7 (62.6, 66.8) |
| 1.2.2 Cancer etiology knowledge | 57.6 (54.9, 60.2) | 51.4 (45.1, 57.5) | 60.2 (57.5, 63.0) |
| 2.2.3 Prevention measures (Others) | 55.9 (53.2, 58.5) | 52.4 (46.1, 58.7) | 57.4 (54.6, 60.1) |
| 5.1.2 The method of cancer pain management | 53.6 (50.9, 56.2) | 50.1 (43.8, 56.5) | 55.1 (52.4, 57.8) |
| 1.1.2 Cancer is preventable and treatable | 43.0 (40.3, 45.7) | 39.8 (33.3, 46.3) | 44.4 (41.7, 47.1) |
| 2.1.4 Risk factors (Infection) | 38.1 (35.5, 40.7) | 32.9 (26.7, 39.1) | 40.3 (37.7, 43.0) |
| 3.3.1 Screening population | 37.1 (34.3, 39.8) | 32.7 (25.8, 39.6) | 39.0 (36.3, 41.6) |
| 2.1.2 Risk factors (Unhealthy life style) | 35.0 (32.4, 37.6) | 31.0 (24.7, 37.2) | 36.8 (34.1, 39.4) |

**Table S3 The** **tertiary indicators rates of cancer literacy in Liaoning Province, China, 2021.**
